# Ticagrelor Use in Acute Myocardial Infarction: Insights From the National Cardiovascular Data Registry

**DOI:** 10.1161/JAHA.117.008125

**Published:** 2018-06-09

**Authors:** Sukhdeep S. Basra, Tracy Y. Wang, DaJuanicia N. Simon, Karen Chiswell, Salim S. Virani, Mahboob Alam, Vijay Nambi, Ali E. Denktas, Anita Deswal, Biykem Bozkurt, Christie M. Ballantyne, Eric D. Peterson, Hani Jneid

**Affiliations:** ^1^ Center for Advanced Heart Failure University of Texas Health Science Center at Houston TX; ^2^ Section of Cardiology Department of Medicine Baylor College of Medicine Houston TX; ^3^ Michael E. DeBakey, Veteran Affairs Medical Center Houston TX; ^4^ Duke Clinical Research Institute Durham NC

**Keywords:** acute myocardial infarction, aspirin, clopidogrel, P2Y_12_, prasugrel, ticagrelor, Health Services, Quality and Outcomes, Statements and Guidelines

## Abstract

**Background:**

Ticagrelor is a P2Y_12_ receptor inhibitor with superior clinical efficacy compared with clopidogrel. However, it is associated with reduced efficacy when combined with a high‐dose aspirin.

**Methods and Results:**

Patients in the acute coronary treatment and intervention outcomes network (ACTION) Registry‐Get With The Guidelines (GWTG) with acute myocardial infarction from October 2013 through December 2014 were included in the study (167 455 patients; 622 sites). We evaluated temporal trends in the prescription of P2Y_12_ inhibitors, and identified factors associated with ticagrelor use at discharge. Among patients discharged on ticagrelor and aspirin (21 262 patients), we evaluated the temporal trends and independent factors associated with high‐dose aspirin prescription at discharge. Ticagrelor prescription at discharge increased significantly from 12% to 16.7% (*P*<0.0001). Decreases in prasugrel and clopidogrel use at discharge (15.7%–13.9% and 54.2%–51.1%, respectively, *P*<0.0001) were also observed. Independent factors associated with preferential ticagrelor prescription at discharge over clopidogrel included younger age, white race, home ticagrelor use, invasive management, and in‐hospital re‐infarction and stroke (*P*<0.0001 for all), whereas older age, female sex, prior stroke, home ticagrelor use, and in‐hospital stroke (*P*<0.0001 for all) were associated with preferential ticagrelor prescription at discharge over prasugrel. High‐dose aspirin was used in 3.1% of patients discharged on ticagrelor. Independent factors associated with high‐dose aspirin prescription at discharge included home aspirin use, diabetes mellitus, previous myocardial infarction, previous coronary artery bypass graft, ST‐segment–elevation myocardial infarction, cardiogenic shock, and geographic region (*P*=0.01).

**Conclusions:**

Our contemporary analysis shows a modest but significant increase in the use of ticagrelor and a high rate of adherence to the use of low‐dose aspirin at discharge.

**Clinical Perspective**

**What Is New?**

- There has been a steady increase in ticagrelor prescription at discharge in patients with acute myocardial infarction.
- Several independent factors predict preferential ticagrelor prescription at discharge over prasugrel or clopidogrel.
- There is a high rate of adherence to the American College of Cardiology/American Heart Association guidelines on the use of low‐dose aspirin in patients treated with ticagrelor.

**What Are the Clinical Implications?**

- Contemporary utilization patterns of P2Y_12_ inhibitors show increasing use of ticagrelor at discharge for acute myocardial infarction, with significant compliance to use of low‐dose aspirin in patients treated with ticagrelor.

## Introduction

Dual antiplatelet therapy is the cornerstone therapeutic strategy in patients with acute myocardial infarction (AMI). Ticagrelor, an oral, direct and reversible, P2Y_12_ receptor antagonist significantly was found to reduce the composite primary end point of vascular death, myocardial infarction, and stroke, without a significant increase in the safety end point of major bleeding, when compared with clopidogrel in the PLATO (platelet inhibition and patient outcomes) trial.^1^ Ticagrelor is currently approved by the US Food and Drug Administration (FDA) for the prevention of atherothrombotic events in adult patients with AMI.^2^ A paucity of data exists on the contemporary patterns of ticagrelor use in real‐world patients with AMI in the United States. The acute coronary treatment and intervention outcomes network (ACTION) Registry‐Get With The Guidelines (GWTG) started collecting data on ticagrelor use in January 2013, and thus offers a unique opportunity to examine patterns of ticagrelor use compared with other antiplatelet agents (clopidogrel and prasugrel) in a nationwide contemporary registry of AMI patients. Additionally, the availability of these data makes it possible to further explore factors associated with the preferential use of ticagrelor over other antiplatelet agents.

A subanalysis from PLATO identified a significant treatment–geographic region interaction (*P*=0.045) and reported a reduced efficacy of ticagrelor versus clopidogrel in North American patients.^3^ A comprehensive systematic analysis independently identified differences in aspirin dosing as the likely reason for the observed interaction.^4^ Thereafter, the US FDA issued a black box warning regarding ticagrelor use, stating that a maintenance dose of aspirin >100 mg daily (high‐dose aspirin) reduced the effectiveness of ticagrelor and should be avoided. The 2016 American College of Cardiology/American Heart Association guidelines on dual antiplatelet therapy recommended the administration of a P2Y_12_ inhibitor (clopidogrel or ticagrelor) in addition to aspirin to all patients with non‐ST‐elevation–myocardial infarction (NSTEMI) in the absence of any contraindications (Class I, Level of Evidence B).^2^ They also stated that it is reasonable to use ticagrelor in preference to clopidogrel in these patients (Class IIa, Level of Evidence B) and recommended the optimal maintenance dose of aspirin to be 81 mg daily in all patients receiving ticagrelor. However, there is currently no data available on the contemporary dosing pattern of aspirin at discharge in patients treated with ticagrelor.

We therefore aimed to examine, from the ACTION Registry‐GWTG database, (1) the current patterns of ticagrelor use (frequency, temporal trends, predictors of use, hospital variability) early (within 24 hours of presentation) and at discharge in patients with AMI and (2) the current patterns of aspirin dosing in AMI patients receiving ticagrelor (frequency, temporal trends, and predictors of prescription of high‐dose aspirin at discharge).

## Methods

The ACTION Registry‐GWTG is a large national quality‐improvement registry, which collects data on consecutive hospitalized patients presenting with STEMI and NSTEMI at participating centers across the Unites States. Antiplatelet agent choice was completely at the discretion of the treating physician. Trained personnel abstracted data from medical records using standardized data definitions as previously reported.^4^ Data abstracted included de‐identified patient demographics, clinical presentation, medications, procedures, and in‐hospital outcomes and were under the oversight of the Duke Clinical Research Institute analytic center's institutional review board. The requirement for informed consent from the study participants was waived because the patient data were de‐identified. The ACTION Registry‐GWTG started capturing information on ticagrelor use in January 2013 in the Data Collection Tool Version 2.3.1. This provided a unique opportunity to assess contemporary patterns of antiplatelet agents use in patients with AMI following the FDA approval of ticagrelor in July 2011.

The data, analytic methods, and study materials will not be made available to other researchers for purposes of reproducing the results or replicating the procedure.

### Study Population

We evaluated patients presenting with AMI between October 10, 2013 and December 31, 2014 to allow for a lead‐in period for complete reporting of data on ticagrelor from all hospitals and stabilization of contemporary trends of prescription of antiplatelet agents across the country. We included all 887 026 patients presenting with AMI at 1083 participating centers across the United States and enrolled in the ACTION Registry‐GWTG registry. We excluded patients who were admitted before October 2013 (563 638 patients), patients transferred out to other hospitals, and patients from hospitals without at least 1 patient discharged on ticagrelor, which might suggest that ticagrelor was not on formulary, which left us with 167 455 patients from 622 participating sites for the analysis on contemporary patterns of use of P2Y_12_ inhibitors. The final patient population for analyzing discharge aspirin dose included 21 262 patients receiving ticagrelor at discharge across 620 participating sites.

### Statistical Analyses

We initially examined temporal trends in the proportions of AMI patients who were treated with ticagrelor early (within 24 hours of first medical contact) and at discharge. This was done using a test for linear trend that modeled the patient's quarter of presentation as an ordinal independent variable using logistic regression modeling, and the analyses were further stratified by the clinical syndrome at presentation (STEMI versus NSTEMI). In patients receiving P2Y_12_ antagonists at discharge, demographic and clinical variables were compared among the ticagrelor‐, prasugrel‐, and clopidogrel‐treated groups. Categorical variables are presented as frequencies and percentages and compared using the χ^2^ test. Continuous variables are presented as medians and their interquartile ranges (Q1–Q3) and compared using the Kruskal–Wallis test.

We thereafter assessed discharge ticagrelor use stratified by predicted risk of in‐hospital bleeding and mortality (ACTION bleeding^5^and mortality^6^ scores, respectively). These scores were previously derived and validated using the ACTION Registry‐GWTG data. Patients were stratified into high‐ and low‐risk groups for bleeding and mortality based on median ACTION bleeding and mortality scores, and the frequency of discharge ticagrelor use was plotted separately for STEMI and NSTEMI patients. For the analyses examining factors associated with the preferential prescription of ticagrelor at discharge over other P2Y_12_ inhibitors, we excluded patients who died during hospitalization (7514 patients), patients discharged to comfort measures or hospice care (2910 patients), and patients not discharged on any P2Y_12_ receptor inhibitor (30 368 patients), leaving a cohort of 126 633 patients from 622 sites for the multivariable adjustment analyses. These analyses were performed to identify independent factors associated with preferential use of ticagrelor over prasugrel (prasugrel reference group) and ticagrelor over clopidogrel (clopidogrel reference group) using the generalized estimating equations logistic regression model with constant correlation between patients within hospitals. Variables included in the model included age, sex, race, insurance status, hypertension, diabetes mellitus, dyslipidemia, current/recent smoker, dialysis, prior stroke, prior myocardial infarction, prior percutaneous coronary intervention, prior coronary artery bypass grafting, prior congestive heart failure, atrial fibrillation/flutter, peripheral arterial disease, home medications, clinical factors at presentation (STEMI/NSTEMI, heart failure, cardiogenic shock, heart rate, systolic blood pressure, baseline hemoglobin, creatinine, troponin, and international normalized ratio [INR]), in‐hospital medications (unfractionated heparin, enoxaparin, bivalirudin, glycoprotein 2B3A inhibitor), in‐hospital clinical events (re‐infarction, cardiogenic shock, heart failure, stroke, cardiac arrest, bleeding event, blood transfusion), and hospital‐related factors (hospital size, region, teaching status). The extent of missing data for variables used in modeling was low (<2%), except for INR value, which was available in only 23% of patients. Variables with missing data were imputed to the median of the nonmissing values for continuous variables and to the most frequently occurring value for categorical variables, except for INR value. Using the indicator variable INR measured (yes or no), a new INR variable was created. When INR was not measured, the new INR variable was imputed to the minimum of the nonmissing INR values and set to the reported INR value when INR was measured. Both variables were included in the model together and the coefficient for the new INR variable is estimated only among those in whom INR was measured. Odds ratio with corresponding 95% confidence intervals (CIs) and *P* values are presented. Hospital‐ and regional‐level variation was evaluated using hierarchical logistic regression modeling with hospital‐specific random intercepts to test for variability in ticagrelor prescription at discharge.

Additionally, we also evaluated temporal trends in high‐dose aspirin prescription at discharge by quarter, and assessed the demographic and clinical factors associated with use of high‐dose aspirin in patients who were discharged on ticagrelor. For the analysis of discharge aspirin dose in patients receiving ticagrelor at discharge, we additionally excluded patients not discharged on ticagrelor (135 268 patients) and patients not discharged on aspirin or with missing discharge aspirin dose (501 patients). A multivariable generalized estimating equations logistic regression model was constructed for the binary outcome of high‐dose aspirin at discharge versus low‐dose aspirin at discharge. Hierarchical logistic regression modeling with hospital‐specific random intercepts was used to describe the variation in the high‐dose aspirin prescription rates between hospitals. All analyses were performed using SAS software, version 9.3 (SAS Institute, Cary, NC). A 2‐sided *P*<0.05 was considered statistically significant.

## Results

### Temporal Trends in Ticagrelor Use

**Figure 1:**
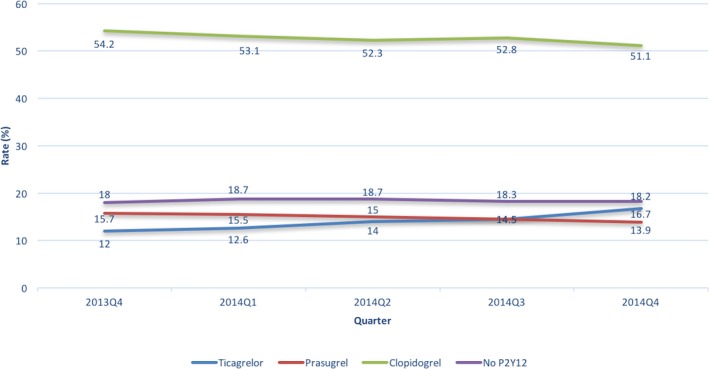
Temporal trends in discharge P2Y_12_ inhibitor use by quarter.

**Figure 2:**
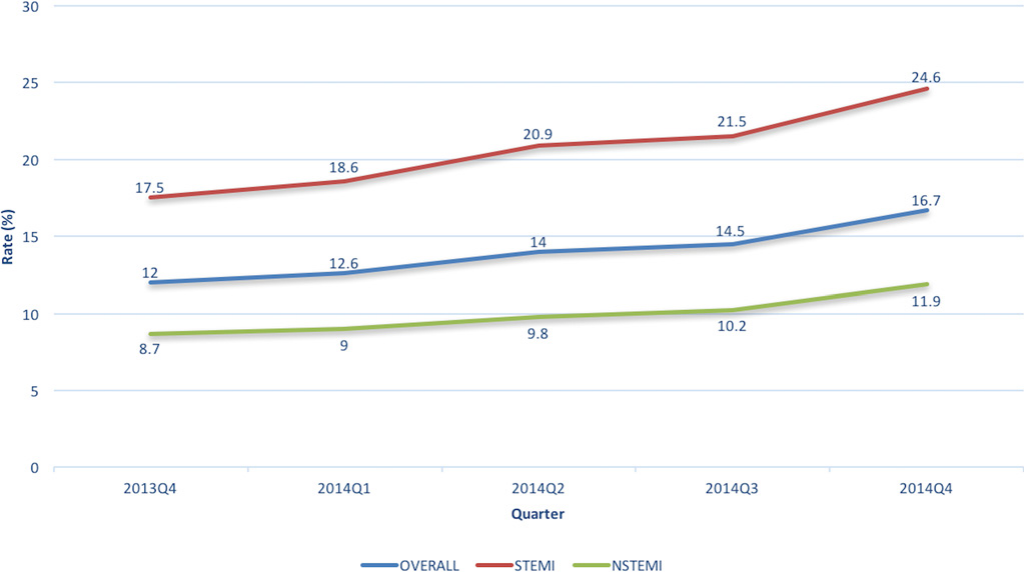
Temporal trends in discharge ticagrelor use by indication. NSTEMI indicates non‐ST‐segment–elevation myocardial infarction; STEMI, ST‐segment–elevation myocardial infarction.

P2Y_12_ inhibitors were prescribed in 68.5% of AMI patients within 24 hours of admission and 82% of patients at discharge. This remained relatively unchanged over time from October 2013 to December 2014 (Figure 1). Early ticagrelor use increased significantly over time from 11.8% to 16.4% (STEMI 19.6%–27.6%; NSTEMI 6.8%–9.5%; *P*<0.0001 for all). Ticagrelor prescription at discharge increased significantly from 12% to 16.7% (STEMI 17.5%–24.6%; NSTEMI 8.7%–11.9%; *P*<0.0001 for both comparison) (Figures 1 and 2). During this time, we observed a significant decrease in the early use of prasugrel (13.3%–11.9%, *P*<0.0001) and clopidogrel (43.4%–39.5%, *P*<0.0001). Similar trends were observed in the use of prasugrel (15.7%–13.9%, *P*<0.0001) and clopidogrel (54.2%–51.1%, *P*<0.0001) at discharge (Figure 1).

### Factors Associated With Ticagrelor Use at Discharge

Baseline characteristics were significantly different between patients who were treated with ticagrelor compared with their clopidogrel and prasugrel counterparts (Table 1). Independent factors associated with ticagrelor prescription over clopidogrel or prasugrel at discharge are summarized in Tables 2 and 3, respectively. Overall, independent factors associated with ticagrelor prescription at discharge over clopidogrel included younger age, white race, private insurance, home ticagrelor use, invasive management and in‐hospital re‐infarction, stroke (*P*<0.0001 for all), and cardiogenic shock (*P*=0.001), whereas prior cerebrovascular accident (CVA), atrial fibrillation, and coronary artery bypass graft surgery favored clopidogrel prescription at discharge. Independent factors associated with ticagrelor prescription at discharge over prasugrel included older age, female sex, prior stroke, home ticagrelor use, in‐hospital stroke (*P*<0.001 for all), and cardiogenic shock (*P*=0.001), whereas prior diabetes mellitus (*P*<0.001) and home prasugrel use (*P*<0.0001) were associated with prasugrel prescription at discharge.

**Table 1:** Baseline Characteristics of Patients With AMI Treated With Antiplatelet Agents Stratified by Discharge Antiplatelet Agent

Variable	Overall (N=126 663)	Discharge Ticagrelor (N=21 763)	Discharge Prasugrel (N=23 130)	Discharge Clopidogrel (N=81 770)	*P* Value
Age, y	63.2±13	62±12.5	57.7±10.4	65.1±13.4	<0.01
Male sex, %	68.1	69.7	75.6	65.6	<0.01
Weight, kg	88.0±21.7	88.4±21.3	92.3±20.8	86.6±22	<0.01
BMI, kg/m^2^	29.8±6.6	29.7±6.5	30.6±6.4	29.5±6.7	<0.01
Medicare, %	22.3	20.3	15.2	24.8	<0.01
HMO/private, %	58.8	62.8	64.0	56.3	<0.01
Hypertension, %	73.2	68.9	66.1	76.4	<0.01
Diabetes mellitus, %	33.4	29.2	29.4	35.7	<0.01
Hyperlipidemia, %	61.7	58.4	57.6	63.7	<0.01
Prior congestive heart failure, %	10.1	6.0	5.1	12.7	<0.01
Prior myocardial infarction, %	25.2	20.4	21.6	27.5	<0.01
Cardiogenic shock, %	2.5	3.1	2.4	2.4	<0.01
Prior PCI, %	27.9	24	25.2	29.7	<0.01
Prior CABG, %	13.4	9.4	8.2	16.0	<0.01
Atrial fibrillation, %	5.4	3.6	2.4	6.7	<0.01
CVA, %	6.9	5.7	1.7	8.7	<0.01
STEMI, %	43.3	56.0	54.8	36.7	<0.01
ACTION mortality score	29.7±9.3	29.6±9.1	27.8±8.5	30.2±9.5	<0.01
ACTION bleeding score	26.8±7.8	26.5±7.6	25.0±7.1	27.4±8.0	<0.01

ACTION indicates acute coronary treatment and intervention outcomes network; AMI, acute myocardial infarction; BMI, body mass index; CABG, coronary artery bypass graft surgery; CVA, cerebrovascular accident; HMO, Health Maintenance Organization; PCI, percutaneous coronary intervention; STEMI, ST‐segment–elevation myocardial infarction.

**Table 2:** Factors Associated With Preferential Use of Ticagrelor Versus Clopidogrel at Discharge

Variable	OR (95% CI)	*P* Value
Age		
Age (per 5‐y increase & ≤70 y)	0.97 (0.96–0.98)	<0.01
Age (per 5‐y increase & >70 y)	0.86 (0.84–0.87)	<0.01
White race vs (nonwhite race)	1.17 (1.12–1.23)	<0.01
Home clopidogrel	0.54 (0.51–0.58)	<0.01
Home ticagrelor	23.03 (18.04–29.39)	<0.01
Home prasugrel	2.17 (1.73–2.73)	<0.01
Home warfarin use	0.61 (0.55–0.68)	<0.01
Prior CHF	0.88 (0.83–0.94)	<0.01
Prior hypertension	0.94 (0.91–0.97)	<0.01
Prior stroke	0.91 (0.85–0.97)	<0.01
Current/recent smoker vs nonsmoker	0.83 (0.80–0.86)	<0.01
Prior CABG	0.87 (0.82–0.91)	<0.01
Prior atrial fibrillation	0.83 (0.76–0.89)	<0.01
Creatinine value (per 1 mg/dL increase)	0.95 (0.93–0.97)	<0.01
Hemoglobin (per 1 g/dL increase)	0.98 (0.97–0.99)	<0.01
Insurance		
Medicare vs (HMO/private)	0.87 (0.84–0.91)	<0.01
Medicaid vs (HMO/private)	0.71 (0.66–0.77)	<0.01
Self vs (HMO/private)	0.63 (0.59–0.68)	<0.01
Other vs (HMO/private)	0.72 (0.65–0.79)	<0.01
STEMI (vs NSTEMI)	2.51 (2.17–2.89)	<0.01
NSTEMI management		
PCI vs medical management	4.27 (3.79–4.82)	<0.01
CABG vs medical management	0.39 (0.29–0.52)	<0.01
In‐hospital RBC/whole blood transfusion	0.62 (0.56–0.69)	<0.01
In‐hospital re‐infarction	1.88 (1.59–2.22)	<0.01
In‐hospital CVA/stroke	0.66 (0.53–0.82)	0.02
In‐hospital access site bleeding	1.18 (1.02–1.35)	0.02
Cardiogenic shock on presentation	1.16 (1.06–1.27)	<0.01
Region		
Northeast vs (South)	1.59 (1.12–2.25)	0.01
West vs (South)	0.59 (0.40–0.86)	<0.01
Midwest vs (South)	1.00 (0.71–1.41)	0.99

CABG indicates coronary artery bypass graft; CHF, congestive heart failure; CI, confidence interval; CVA, cerebrovascular accident; HMO, Health Maintenance Organization; NSTEMI, non‐ST‐segment–elevation myocardial infarction; OR, odds ratio; PCI, percutaneous coronary intervention; RBC, red blood cell; STEMI, ST‐segment–elevation myocardial infarction.

**Table 3:** Factors Associated With Preferential Use of Ticagrelor Versus Prasugrel at Discharge

Variable	OR (95% CI)	*P* Value
Age		
Age (per 5‐y increase & ≤70 y)	1.05 (1.04–1.06)	<0.01
Age (per 5‐y increase & >70‐y)	1.75 (1.65–1.85)	<0.01
Home ticagrelor	4.62 (3.81–5.60)	<0.01
Home prasugrel	0.12 (0.10–0.14)	<0.01
Home aspirin	1.06 (1.02–1.10)	<0.01
Home β‐blockers	1.05 (1.00–1.10)	0.03
Prior stroke	2.76 (2.40–3.17)	<0.01
Female sex	1.14 (1.10–1.19)	<0.01
Event: stroke	3.13 (2.02–4.87)	<0.01
Prior diabetes mellitus	0.93 (0.89–0.97)	<0.05
Prior PCI	0.93 (0.88–0.98)	<0.01
Prior MI	0.93 (0.88–0.98)	<0.01
Cardiogenic shock on presentation	1.19 (1.07–1.32)	<0.01
Acute glycoprotein IIb/IIIa inhibitor use (vs none)	0.87 (0.82–0.93)	<0.01
STEMI (vs NSTEMI)	0.74 (0.63–0.87)	<0.01
Region		
Northeast vs (South)	1.41 (0.97–2.05)	0.06
West vs (South)	1.01 (0.75–1.35)	0.95
Midwest vs (South)	1.51 (1.11–2.05)	0.01
INR (per 1‐unit increase)	1.06 (1.01–1.11)	0.02

CI indicates confidence interval; INR, international normalized ratio; MI, myocardial infarction; NSTEMI, non‐ST‐segment–elevation myocardial infarction; OR, odds ratio; PCI, percutaneous coronary intervention; STEMI, ST‐segment–elevation myocardial infarction.

**Figure 3:**
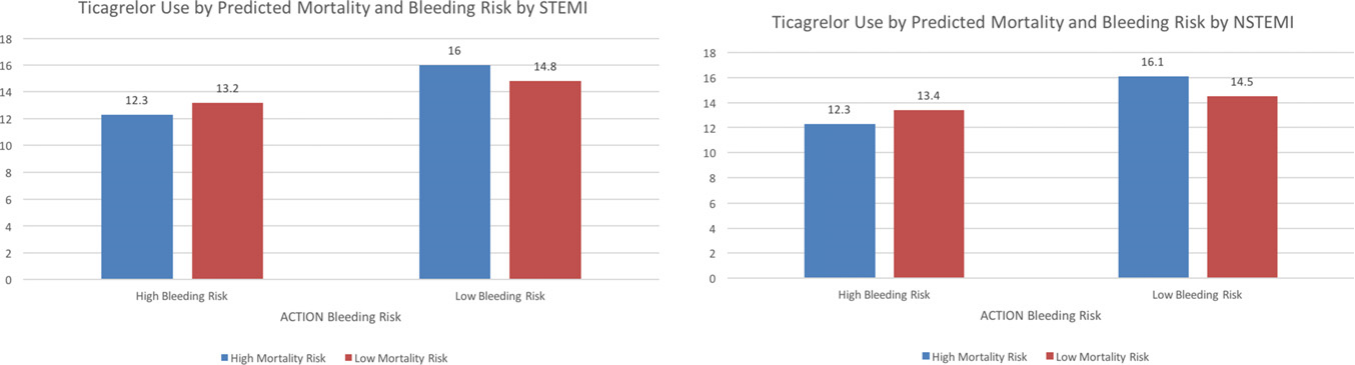
Discharge ticagrelor use by predicted mortality and bleeding risk. ACTION indicates acute coronary treatment and intervention outcomes network; NSTEMI, non‐ST‐segment–elevation myocardial infarction; STEMI, ST‐segment–elevation myocardial infarction.

Ticagrelor use decreased with increased risk of mortality (*P*=0.0002 for trend) and increased risk of bleeding (*P*<0.0001 for trend) based on the ACTION mortality score and ACTION bleeding score. When stratified by STEMI/NSTEMI presentation, the highest utilization of ticagrelor was noted in patients with high risk of mortality and low risk of bleeding (Figure 3).

A significant hospital‐ and regional‐level variability in ticagrelor use at discharge was observed (*P*<0.0001 for both). Patients were most likely to be discharged on ticagrelor in the Northeast (20.6%, 95% CI, 19.9%–21.2%), followed by Midwest (14.9%, 95% CI, 14.6%–15.2%), South (13.3%, 95% CI, 13.0–13.5%), and West (10.2%, 95% CI, 9.8–10.7%) with a median percentage of use of 12.2% (5.3, 95% CI, –22.7%) across the 622 sites across the country.

### Temporal Trends, Variability, and Factors Associated With the Use of High‐Dose Aspirin at Discharge

**Figure 4:**
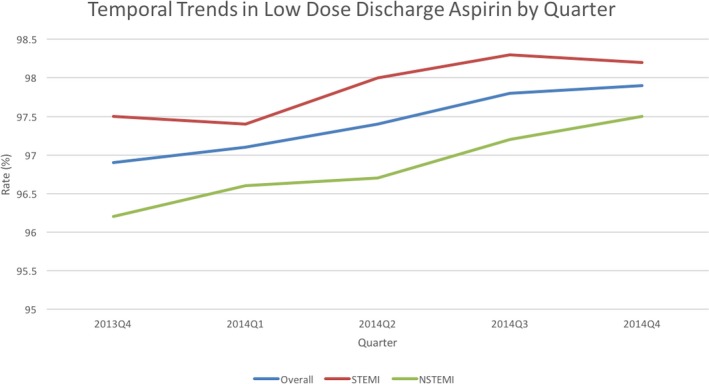
Temporal trends in low‐dose aspirin use in patients discharged on ticagrelor and aspirin stratified by indication. NSTEMI indicates non‐ST‐segment–elevation myocardial infarction; STEMI, ST‐segment–elevation myocardial infarction.

Of 21 262 patients receiving ticagrelor at discharge, only 538 (2.5%) patients (2.5% overall, STEMI 2.1%, NSTEMI 3.1%) were discharged on high‐dose aspirin. The proportion of patients receiving ticagrelor who were discharged on high‐dose aspirin diminished minimally but significantly over time (from 3.1% in 2013 Q4 to 2.1% in 2014 Q4, *P*<0.0001 for the trend). High‐dose aspirin use was much higher in patients discharged on prasugrel (29.5%) and clopidogrel (28.2%) as compared with ticagrelor (2.5%) for the same time period (Figure 4).

Patients discharged on high‐dose aspirin were older, with a higher prevalence of comorbidities including hypertension, diabetes mellitus, hyperlipidemia, peripheral arterial disease, myocardial infarction, prior percutaneous coronary intervention, prior coronary artery bypass graft surgery, and had more likelihood of being treated at home with aspirin, clopidogrel, ticagrelor, and statins before presentation (Table 4). Patients presenting to academic hospitals as well as those presenting with STEMI were less likely to be discharged on high‐dose aspirin. There were no significant differences in ACTION mortality and bleeding scores among patients discharged on high‐ versus low‐dose aspirin. After multivariable adjustment, independent factors associated with high aspirin dose at discharge included home aspirin use, diabetes mellitus, previous MI, previous coronary artery bypass graft surgery, NSTEMI, and regional variability (*P*=0.01). Clinical presentation as STEMI and as cardiogenic shock were independently associated with low‐dose aspirin at discharge (Table 5). Significant region‐level (Northeast 1.6% [1.2%–2.1%], Midwest 2.1% [1.8%–2.5%], South 2.9% [2.6%–3.2%], West 3.4% [2.7%–4.3%], *P*<0.001) and hospital‐level (*P*=0.001) variation was noted in the prescription of high‐dose aspirin at discharge.

**Table 4:** Baseline Characteristics of Patients Treated With Ticagrelor Stratified by Discharge Aspirin Dose

Variable	Overall (N=21 262)	Low‐Dose Aspirin (N=20 724)	High‐Dose Aspirin (N=538)	*P* Value
Age (y) ±SD	61.9±12.4	61.9±12.4	63±12.4	0.02
Male sex, %	69.9	69.9	69.1	0.71
BMI (kg/m^2^) ±SD	29.7±6.4	29.7±6.4	30.0±6.3	0.26
Race				0.39
White, %	80.9	80.9	79.7	
Black, %	10.0	9.9	11.9	
Asian, %	2.3	2.4	1.5	
Hispanic, %	5.8	5.8	6.3	
Others, %	0.6	0.6	0.6	
Insurance				0.36
HMO/private, %	62.9	63.0	60.6	
Medicare, %	20.1	20.0	23.1	
Medicaid, %	4.7	4.8	4.3	
Military/VAMC, %	1.3	1.3	1.9	
Self/none, %	10.1	10.1	9.9	
Hypertension, %	68.7	68.5	76.6	<0.01
Diabetes mellitus, %	29.1	28.8	39.4	<0.01
Hyperlipidemia, %	58.3	58.1	67.8	<0.01
Chronic lung disease, %	9.6	9.6	11.7	0.09
Congestive heart failure, %	5.9	5.9	7.3	0.18
Myocardial infarction, %	20.3	20.0	32.2	<0.01
Currently on dialysis, %	1.2	1.2	1.9	0.16
Prior PCI, %	23.9	23.6	36.1	<0.01
Prior CABG, %	9.3	9.1	17.5	<0.01
Atrial fibrillation, %	3.5	3.4	4.3	0.28
CVA, %	5.6	5.6	7.3	0.08
Peripheral arterial disease, %	6.2	6.1	11.2	<0.01
STEMI, %	56.1	56.4	46.3	<0.01
Cardiogenic shock, %	3	3.1	2.4	0.39
Cardiac arrest, %	3.5	3.5	3.2	0.64
Initial hemoglobin (mg/dL) ±SD	14.3±1.9	14.3±1.9	14.0±1.9	<0.01
Academic hospital, %	26.5	26.6	20.6	<0.01
Home medications				
Aspirin	39.3	38.8	56.1	<0.01
Clopidogrel	7.5	7.3	13.2	<0.01
Prasugrel	0.6	0.6	0.9	0.28
Ticagrelor	3.9	3.8	6.3	0.01
Warfarin	2.0	2.0	1.5	0.39
Statin	35.8	35.5	48.0	<0.01
Length of stay (d) ±SD	3.4±3.2	3.4±3.2	3.6±4.0	0.25
ACTION Mortality Score ±SD	29.6±9.1	29.6±9.0	29.4±9.4	0.65
ACTION Bleeding Score ±SD	26.4±7.5	26.4±7.5	27.0±7.5	0.10

ACTION indicates acute coronary treatment and intervention; BMI, body mass index; CABG, coronary artery bypass graft; CVA, cerebrovascular accident; HMO, Health Maintenance Organization; PCI, percutaneous coronary intervention; STEMI, ST‐segment–elevation myocardial infarction; VAMC, Veteran Affairs Medical Center.

**Table 5:** Factors Associated With Preferential Use of High‐Dose Aspirin Versus Low‐Dose Aspirin at Discharge

Variable	OR (95% CI)	*P* Value
Home aspirin use	1.52 (1.29–1.80)	<0.01
Northeast vs (South)	0.53 (0.34–0.85)	<0.01
West vs (South)	1.06 (0.75–1.51)	0.75
Midwest vs (South)	0.72 (0.51–1.00)	0.05
History of diabetes mellitus	1.28 (1.09–1.51)	<0.01
Prior myocardial infarction	1.27 (1.05–1.53)	<0.01
Prior CABG	1.32 (1.05–1.66)	0.01
STEMI (vs NSTEMI)	0.85 (0.73–0.99)	0.03
In‐hospital cardiogenic shock	0.57 (0.33–0.99)	0.04

CABG indicates coronary artery bypass graft; CI, confidence interval; NSTEMI, non‐ST‐segment–elevation myocardial infarction; OR, odds ratio; STEMI, ST‐segment–elevation myocardial infarction.

## Discussion

Our large and contemporary national data set of patients being admitted with AMI shows a modest increase over time in the use of ticagrelor during the early hospitalization period and at discharge, especially in patients presenting with STEMI. We also report several key clinical and demographic factors associated with the preferential use of ticagrelor over clopidogrel or prasugrel at discharge. In addition, we note a very high rate of adherence to the FDA‐recommended use of low‐dose aspirin at discharge in AMI patients treated with ticagrelor. Finally, we also show significant hospital‐level and regional variability in the contemporary prescription patterns of aspirin dose at discharge in these patients, which underscores potential opportunities for care improvement.

Data from the Swedish registries from 2009 to 2013 in 1 04 012 patients showed that ticagrelor was the preferred antiplatelet agent of choice in patients who had acute coronary syndromes and who underwent angiography (ticagrelor 54%, clopidogrel 26%, prasugrel 1%, and no P2Y_12_ inhibitor 18%).^7^ Similar to the trend in our report, data analyzed from Australia from 2009 to 2013 show that the majority of patients were treated with clopidogrel (72%) in preference to prasugrel (14%) and ticagrelor (14%), albeit with increasing use of ticagrelor towards the end of 2013. Additionally, patients treated with ticagrelor were younger, had fewer comorbidities and more likely presented with STEMI compared with their clopidogrel counterparts, with no intergroup differences in 30‐day mortality, major adverse cardiovascular events, and in‐hospital bleeding rates.^8^

There are limited data on the contemporary patterns of use of P2Y_12_ inhibitors in clinical practice in the United States since the introduction of ticagrelor. Data from the Cerner health database on 37 964 patients presenting with STEMI to 77 hospitals (January 2008–June 2013) and undergoing diagnostic coronary angiography and/or percutaneous coronary interventions showed a 77% rate of pretreatment with clopidogrel, compared with 13% and 10% rates for prasugrel and ticagrelor, respectively.^9^ However, this study was limited by the fact that only patients undergoing coronary angiography and P2Y_12_ inhibitors administered as pretreatment were included in the analyses. Data from the Blue Cross Blue Shield of Michigan records of 64 600 patients undergoing percutaneous coronary intervention at 47 Michigan hospitals from January 2012 to March 2014 showed that 72% of patients received clopidogrel, 20% received prasugrel, and only 8% received ticagrelor at discharge, with use of ticagrelor increasing over time. Similar to our study, ticagrelor use was more often noted in patients presenting with STEMI (24.4% versus 18.8%) and cardiogenic shock (1.3% versus 0.9%). However, the study was limited to only those patients who underwent percutaneous coronary intervention, and the results are unlikely to be generalizable to the entire country given the significant regional variability noted in our analysis. Even within Michigan, the variability of ticagrelor prescription between hospitals ranged from 0.5% to 64.9% of hospital discharges, which corroborates the finding of high hospital‐level variability observed in our study.^10^ Analysis from the ACTION Registry‐GWTG in 100 228 STEMI and 158 492 NSTEMI patients between October 2009 and September 2012 shows an increase in prasugrel uptake from 3% to 18% over time. However, this study was conducted before the National Cardiovascular Data Registry ACTION Registry‐GWTG started collecting information on ticagrelor use in January 2013. Additionally, generic versions of clopidogrel were approved in May 2012, likely altering the value of care and potentially affecting the patterns of prescription of P2Y_12_ inhibitors. Data from 1717 patients from 3 centers in Spain between February 2014 and to December 2015 show a progressive increase in ticagrelor prescription from 15% to 28% in patients presenting with acute coronary syndromes. Similar to our study, the authors noted that patients treated with ticagrelor were more likely to be younger, present with STEMI, and have lesser comorbidities.^11^ Our study represents the largest nationwide US data set of patients treated with ticagrelor and includes all patients presenting with AMI irrespective of the management strategy. It also includes data on both early use and discharge prescription of ticagrelor and patients treated after the generic availability of clopidogrel, and thus summarizes the most contemporary practice patterns of P2Y_12_ inhibitor use in the United States.

We also demonstrated higher use of ticagrelor at discharge in patients initially presenting with STEMI (*P*<0.0001) and cardiogenic shock (*P*<0.0001). Overall the ACTION Mortality score and ACTION Bleeding score were noted to be higher for patients treated with ticagrelor as compared with prasugrel and similar between ticagrelor and clopidogrel. Ticagrelor use was noted to be the highest in those with high risk of mortality and low risk of bleeding. This is in concordance with the improved ischemic outcomes and higher risk of non–coronary artery bypass graft surgery–related major bleeding seen in the PLATO trial^1^ as well as current guidelines, which advocate intensive medical therapy in patients with moderate‐ to high‐risk features on presentation to help achieve the greatest ischemic benefit while minimizing bleeding complications.^12^

In our analysis, discharge ticagrelor use in a “real‐world” setting was favored over clopidogrel in younger patients with STEMI, in‐hospital re‐infarction, as well as those who sustained an AMI while already being treated with a potent P2Y_12_ inhibitor like prasugrel. Clopidogrel was favored over ticagrelor in patients with atrial fibrillation, home warfarin therapy, prior CVA, in‐hospital CVA, as well as in those receiving transfusions during hospitalization. This likely reflects practices to reduce bleeding risk in patients at higher risk of bleeding complications. Similarly, ticagrelor was favored over prasugrel in patients who were older, females, had prior stroke or sustained an in‐hospital CVA, which is likely because of the contraindications associated with use of prasugrel (previous transient ischemic attack/CVA, age >75 years, weight <60 kg).^13^ Additionally, patients with diabetes mellitus were more likely to be prescribed prasugrel over ticagrelor based on greater clinical efficacy of prasugrel in diabetic as compared with nondiabetic patients.^14^ Our findings suggest that there is a vast array of factors coming into play in the complex decision making involved in the selection of the appropriate P2Y_12_ inhibitors. These include individualized patient risk–benefit analyses based on ischemic and bleeding hazards, as well as physicians and patients’ preferences, comorbidities, insurance status, home medications, and in‐hospital events.

Our article also shows a high compliance rate with the American College of Cardiology/American Heart Association guidelines and FDA recommendation for the use of low aspirin dose (″≤100 mg daily) in patients receiving ticagrelor. In the PLATO trial, concomitant use of high‐dose aspirin with ticagrelor was noted in 53.6% of patients in North America compared with 1.7% patients in the rest of the world.^1^ This was hypothesized to be the main reason for the impaired efficacy and higher bleeding rate noted in patients in North America compared with the rest of the world, and was confirmed using Cox proportional regression and landmark analyses after evaluating 36 potential factors that could account for the observed geographical variation. This work by Mahaffey et al formed the basis for the FDA black box warning on discharge aspirin dosing in patients receiving ticagrelor.^3^ Nevertheless, there is currently a paucity of data describing the real‐world practice patterns of discharge aspirin regimen in the United States, and our report represents one of the first and largest analyses on this topic. Our data are very encouraging because they show that most patients are being discharged on aspirin dose ≤ 100 mg daily, and this continues to improve over time (96.9% in 2013 to 97.9% in 2014). We also noted significant hospital‐level variability in prescription of high‐dose aspirin with ticagrelor. This suggests that local factors and operator preference may be likely reasons for these inappropriate prescription patterns and highlight the existing opportunities to improve care. Recent data from the National Cardiovascular Data Registry shows that between 2007 and 2011, 60.5% patients with AMI were still treated with high‐dose aspirin.^15^ With the recent changes in guideline recommendations, there has been a gradual shift to the use of low‐dose aspirin at discharge in patients with AMI. In our study, high‐dose aspirin was used in 29.5% of patients discharged on prasugrel and 28.2% of patients discharged on clopidogrel during the same time period. This reflects a broader acceptance and compliance with the guidelines and warnings specific to discharge aspirin dosing with ticagrelor.

Our study has several limitations. First, participation in the registry is voluntary and participating centers tend to be larger tertiary care centers, which differ from practice patterns in community hospitals. Second, only information during the index hospitalization visit is reported and data on subsequent clinical follow‐up, long‐term outcomes, and changes to P2Y_12_ inhibitors are not available. Additionally, the rationale and appropriateness for the selection of the various P2Y_12_ inhibitors are not captured and cannot be ascertained in the ACTION Registry‐GWTG registry. Furthermore, data are abstracted retrospectively by trained chart abstractors using standardized definitions and thus are reliant on accurate chart abstraction. Finally, given the observational nature of the data and the inability to adjust for unmeasurable confounders, our report established associations rather than causality.

## Conclusions

Our contemporary report shows a modest but significant increase in the use of ticagrelor early and at discharge, with simultaneous decline in the use of clopidogrel and prasugrel in patients presenting with AMI. We also demonstrate a high rate of adherence to the FDA recommendation and the American College of Cardiology/American Heart Association guidelines with respect to the use of low‐dose aspirin at discharge in patients with AMI treated with ticagrelor, and highlight significant regional and hospital variability in ticagrelor prescription and aspirin dose at discharge. The latter represent important opportunities for future improvements in care of patients with AMI.

## Sources of Funding

This research was supported by the American College of Cardiology Foundation's National Cardiovascular Data Registry (NCDR). The views expressed in this presentation represent those of the author(s), and do not necessarily represent the official views of the NCDR or its associated professional societies identified at http://CVQuality.ACC.org/NCDR. For more information go to http://CVQuality.ACC.org/NCDR or email ncdrresearch@acc.org.

## Disclosures

Dr Ballantyne reports having received consultant fees/honoraria from Abbott Diagnostic, Amarin, Amgen, AstraZeneca, Eli Lilly, Esperion, Genzyme, Ionis, Matinas BioPharma Inc, Merck & Company, Novartis, Pfizer, Regeneron, Roche Diagnostic, Sanofi‐Synthelabo; other—Roche; research/research grants—Abbott Diagnostic, Amarin, Amgen, Eli Lilly, Esperion, Novartis, Otsuka, Pfizer, Regeneron, Roche Diagnostic, Sanofi‐Synthelabo, and Takeda. Dr Peterson reports receiving consultant fees/honoraria from Astra Zeneca, Bayer, Boehringer Ingelheim, Genentech, Janssen, Merck & Co., Inc, Sanofi, Valeant Pharmaceuticals International; research/research grants—Janssen. Dr Wang received consultant fees/honoraria from Astra Zeneca, Eli Lily, Merck, Premier, Inc; research/research grants—AstraZeneca, Boston Scientific, Bristol‐Myers Squibb Company, Eli Lilly/Daiichi Sankyo Alliance, Gilead, Regeneron. Dr Nambi received research grants from Roche Diagnostics/Baylor College of Medicine, General Electric, and TomTec. Dr Alam is on the speakers bureau with Jannssen. The remaining authors have no disclosures to report.
